# How do psychosocial determinants in migrant women in the Netherlands differ from these among their counterparts in their country of origin? A cross-sectional study

**DOI:** 10.1186/1471-2458-11-397

**Published:** 2011-05-26

**Authors:** Vera Nierkens, Maya V van der Ploeg, Marja Y van Eer, Karien Stronks

**Affiliations:** 1 Department of Social Medicine, Academic Medical Center - University of Amsterdam P.O. Box 22700, 1100 DE Amsterdam, the Netherlands; 2 Department of Internal Medicine, Diakonessenhuis, Paramaribo, Suriname

## Abstract

**Background:**

Migration of non-Western women into Western countries often results in an increase in smoking prevalence among migrant women. To gain more insight into how to prevent this increase, we compared psychosocial determinants of smoking between Surinamese women in Suriname and those in the Netherlands.

**Methods:**

Data were obtained between 2000 and 2004 from two cross-sectional studies, the CVRFO study in Suriname (n = 702) and the SUNSET study in the Netherlands (n = 674). For analyses of determinants, we collected additional data in CVRFO study population (n = 85). Differences between the two groups were analysed by chi-square analyses and logistic regression analyses.

**Results:**

As was found in other studies among migrant women, more Surinamese migrant women in the Netherlands smoked (31%) than women in Suriname (16%). More Surinamese women in the Netherlands than in Suriname had a positive affective and cognitive attitude towards smoking (OR = 2.6 (95%CI 1.05;6.39) and OR = 3.3 (95%CI 1.31;8.41)). They perceived a positive norm within their partners and friends regarding smoking more frequently (OR = 6.5 (95%CI 2.7;15.6) and OR = 3.3 (95%CI 1.50;7.25)).

**Conclusion:**

Migrant women are more positive towards smoking and perceived a more positive norm towards smoking when compared with women in the country of origin. Interventions targeted at the psychosocial determinants regarding smoking for newly migrated women, in particular the consequences of smoking and the norm towards smoking might help to prevent an increase in smoking in those populations.

## Background

Smoking remains one of the major causes of cardiovascular diseases and several types of cancer. Seen as a direct health consequence of smoking, these diseases (and others) can be avoided by preventing people from smoking [1]. In Western countries, anti-smoking activities and smoking cessation programs have proved to be a valuable contribution to considerably decreasing the prevalence of smoking [2]. However, the increase in migration of non-Western populations into Western countries poses new challenges in the planning and development of smoking prevention programs [3].

In migrant populations, a multitude of determinants underlie smoking behaviour. These include broader contextual issues, socioeconomic inequalities compared to host populations, psychosocial aspects such as stress, hardship and discrimination, all of which are known to contribute to health inequalities. Finally, socio-cultural factors governing attitudes towards smoking and the process of acculturation to a 'Western' environment are also relevant [4-9]. These processes and their interaction often imply a change in smoking behaviour after migration. As far as smoking in female migrants is concerned, coming from an environment where smoking rates are low and migrating to an environment where smoking is more common, it often imply an increase in smoking [6,10-13]. In the Netherlands this seems to take place in female migrants from Surinam, a former Dutch colony. People from Surinam (who consist mainly of people with a South Asian or African background) comprise the second largest migrant population in the Netherlands and there are indications that the smoking prevalence is higher in the Netherlands compared to the country of origin [3,14].

In order to develop interventions for preventing an increase in smoking among migrant women, insight into the underlying determinants is needed. This includes insight into changes in proximal factors such as psychosocial determinants: attitudes towards smoking, social influences regarding smoking and self-efficacy [15,16]. At present there is limited information on these underlying determinants and how they change with migration, hampering the development of appropriate interventions for migrant women. For example, when women appeared to have a more positive attitude towards smoking after migration, interventions might be targeted to prevent this change in attitude from taking place.

In our study we had the opportunity to compare the determinants of smoking of Surinamese women in Surinam with Surinamese women in the host country, the Netherlands. Our aim was to gain insight into the differences in psychosocial determinants of Surinamese women in the Netherlands compared to those in the country of origin.

## Methods

### Study populations

**Figure 1:**
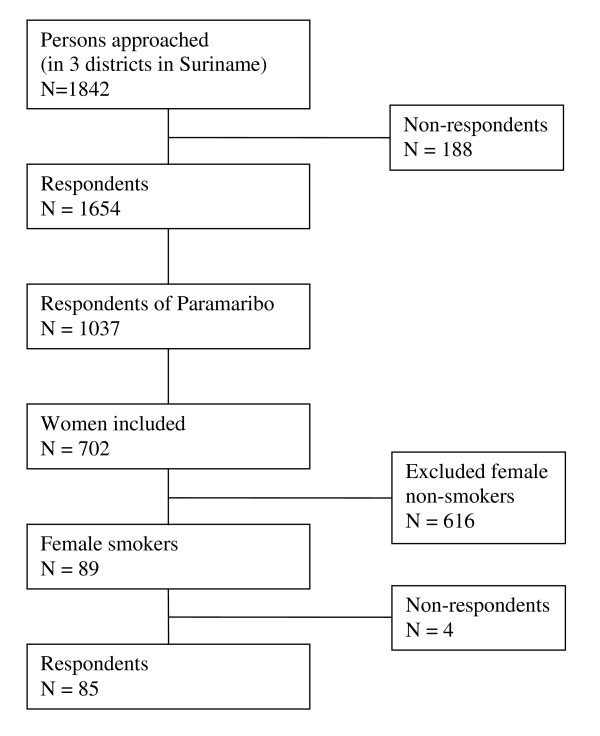
**Flow chart of the CVRFO study**.

**Figure 2:**
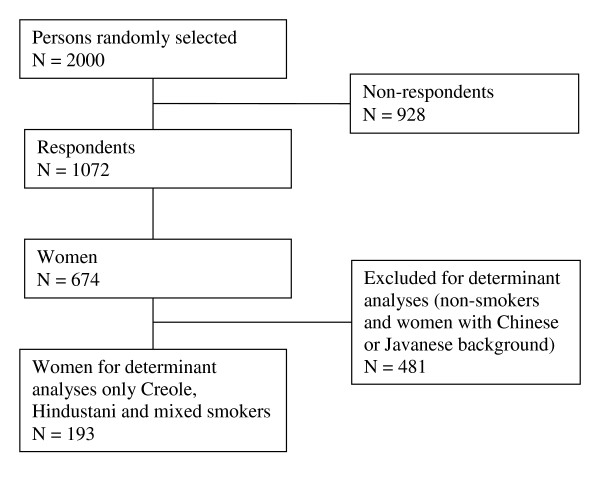
**Flow chart of the SUNSET study**.

Data were obtained from two cross-sectional studies, the CVRFO-study (Cardiovascular risk factor study (CardioVasculair RisicoFactor Onderzoek), in Suriname under supervision of the foundation of the advancement of Scientific Investigation Suriname; sample 1) and the SUNSET study (Surinamese people in the Netherlands, Study of health and ethnicity [SUrinamers in Nederland, Studie naar gezondheid en Etniciteit], AMC, Amsterdam, The Netherlands; sample 2), carried out between 2000 and 2003. Only data from women were used (see also Figures 1 and 2).

The CVRFO-study was carried out in three districts of Suriname from 2000-2001 and was aimed at assessing the cardiovascular risk profile of the Surinamese population aged 15 - 55 years. The CVRFO-study used the method of area sampling, because no reliable sampling frames were available in Suriname. Area sampling occurs by a multistage sampling technique adapted for Suriname [17]. The country was divided into several areas. Equal opportunity for selection was guaranteed for all housing units in the individual areas. A total of 1,842 persons were approached of whom 1,654 participated (response rate 89.8%). Due to practical reasons the additional study was conducted in the district of Paramaribo only. Therefore, only the data from the district of Paramaribo were included (n = 1037) in this study.

The aim of the SUNSET study [18] which was conducted from 2001-2003 in Amsterdam, the Netherlands was to gain insight into the cardiovascular risk profile of the Surinamese population in the Netherlands. An a-select sample of 2,000 persons was drawn from 35-60 year-old, non-institutionalized Surinamese people, living in Amsterdam, the capital city of the Netherlands. If a person could not be contacted at three different times, he was categorized as non-respondent. The net response rate was 62.2% (1,072 persons). Information on the Surinamese population is presented in text box 1.

The assessment of the smoking prevalence was based on the original databases. All women in both samples were included (n = 702 in sample 1, and n = 674 in sample 2). Data were used from the original data collection. To gain insight into the determinants of smoking behaviour, additional data were collected among smokers from the original study in sample 1 in 2003, in order to assess differences in beliefs among women in Suriname and women in the Netherlands. We selected female smokers from the largest ethnic groups - women with a Creole, 'mixed' and Hindustani background (76% of all female smokers) in order to make these data comparable with the data of the SUNSET study (sample 2). In sample 1, ethnicity was based on self-definition. In sample 2 the ethnicity of all four grandparents were asked and those who had at least 75% common in ethnic background, were assigned accordingly: the remainder was assigned as 'mixed' [19].

In sample 1, of the 89 female smokers, 85 were willing to participate in this extra study (net response rate 95.5%). In sample 2, the data on the determinants of smoking were available for 193 women.

### Data collection

The data for the CVRFO main study (sample 1) were collected by trained volunteers in a structured interview and physical examination held in a research location. Additional data about the women's beliefs were collected by structured telephone interviews (N = 42), conducted by one of the authors (MVvdP) as long as the respondents could speak fluent Dutch. If the respondent was not fluent in Dutch, the author carried out a face-to-face interview with an interpreter present (N = 43) (same interpreter for all interviews). Data for the SUNSET study (sample 2) were collected by structured face-to-face interviews during home visits by trained interviewers of the same ethnic background [3].

In both studies, ethical approval was obtained via the regular procedures during the time of the study. All participants provided a written informed consent.

### Measures and questionnaire

To assess smoking prevalence (research question 1) data from both original studies were used.

*Smoking behaviour *was measured by the question 'Do you smoke cigarettes?' in sample 1 and by the question 'Do you smoke?' in sample 2. In this sample respondents were classified as smokers, ex-smokers and non-smokers according to WHO-standards [1].

The questionnaire used in both studies included questions on two important psychosocial determinants: attitude and social influences towards smoking. Questions were developed on the basis of the results of focus group sessions and a review of the most salient beliefs for smoking and smoking cessation among the ethnic Dutch population [20]. *Attitude towards smoking *can be regarded as an expected outcome associated with a particular behaviour. Cognitive and emotional advantages and disadvantages were measured by fourteen beliefs about the perceived consequences of smoking, for example,' If I smoke I have a higher risk of getting heart disease' and, 'If I smoke I think that's normal'. Using principle component analyses (varimax rotation) and reliability analyses, two sub-scales were constructed: emotional attitude towards smoking (α = 0.66, 4 items) and cognitive attitude towards the health consequences of smoking (α = 0.68, 3 items). Five items did not load on any factor and were excluded from the scales.

*Social influences *are defined as the norms that a person perceives towards a behaviour, and the perception of others who carry out that behaviour (perceived behaviour). Subjective norms were assessed by items about judgments made towards the respondent's smoking behaviour by the respondents' partner, close female family and friends, (e.g. If you smoke, what do the women in your close family think about that?). Perceived behaviour was measured by items about the same persons mentioned above (e.g. How many of your female friends smoke?). Scales for the items regarding close family and friends were constructed separately. The internal consistencies of the scales in regard to 'subjective norm of the family', 'subjective norm of friends', 'perceived behaviour of 'family' and 'perceived behaviour of friends' were 0.81, 0.77, 0.69 and 0.73 (2 items) respectively.

Background characteristics were obtained from the original studies and included age (in years), educational level, marital status, financial status and number of people in the household. In both studies, *educational level *was indicated by the highest educational level attained. Four categories were used (none or primary education, lower or general vocational education, intermediate or higher general education or intermediate vocational training and, higher vocational training or university). *Financial status *was assessed through answers to the question: "What is your current financial situation?" with options of - running up debts, using savings, just managing, have some savings, have a lot of savings. In addition, age in which first cigarette was smoked was measured in the SUNSET study and the additional study of the CVRFO study. In the SUNSET study, years since migration and ethnicity of friends was measured.

### Analysis

Smoking prevalence in both datasets was computed with standardization for numbers of respondents per ethnicity (research question 1).

Analyses of determinants of smoking were conducted among female smokers only. We compared smoking related beliefs between the two samples. Firstly, all separate beliefs were dichotomized and the scores were compared between countries and ethnicity. Differences between ethnicities or countries were considered statistically significant at p < 0.01 to adjust for multiple testing [21]. Secondly, logistic analyses were performed with dichotomized scales for attitudes and social influences, adjusted for background characteristics; i.e. ethnicity, age, marital status, organization of the household, education, financial status and religion [22]. Only those background characteristics that appeared to differ significantly (p < 0.25) between the women in Suriname and the Netherlands were included in the analyses.

## Results

### Characteristics

The samples differed significantly in their ethnic composition. In both samples, the percentage of Creole women was the highest of all subpopulations, but in the Dutch sample, more than half had a Creole background (Table 1). The standardized smoking prevalence differed significantly between both samples. In the Netherlands, the prevalence of migrant Surinamese smokers was twice of that in Suriname; 30.9% (of 674; n = 208) of the Surinamese migrant women smoked in the Netherlands compared to 15.5% (of 702; n = 109) in Suriname.

**Table 1:** Percentages of ethnic subpopulations from the SUNSET and CVRFO samples

	Suriname	Netherlands
	N = 702 (%)	N = 674 (%)

Ethnicity		

Hindustani	199 (28.3)	164 (24.3)

Creole	241 (34.3)	387 (57.4) **

Indonesian	85 (12.1)	22 (3.3)

Chinese	7 (1.0)	6 (0.9)

Other	170 (24.2)	85 (14.4)

**p < 0.001

Of the smokers in both samples, we found that the mean age was five years higher in the Dutch sample (Table 2). The educational level of these smokers was higher in the sample from the SUNSET study than in the women in the Surinamese sample; in particular, relatively more women in the Netherlands had a lower general and intermediate vocational training. In both groups of smokers, most of the women were religious. No statistically significant differences were found regarding the mean age at which the first cigarette was smoked. Regarding the sample of the SUNSET study, about half of the women have migrated 26 years or more and most women had mainly friends with a Surinamese background.

**Table 2:** Characteristics of female smokers in the CVRFO and SUNSET samples

		Suriname (CVFRO study)	Netherlands (SUNSET study)
		N = 85 (%)	N = 196 (%)

Religion			

	Hinduism	16 (18.8)	16 (8.4) *

	Islam	7 (8.2)	6 (3.1)

	Christian	62 (72.9)	169 (86.2)

	Missing	0 (0)	5 (2.6)

Education			

	Max. primary education	28 (32.9)	21 (10.7) **

	Low vocational training/lower secondary education	36 (42.4)	86 (43.9)

	Intermediate vocational training/higher secondary education	10 (11.8)	57 (29.1)

	Higher vocational training and University degree	7 (8.2)	26 (13.3)

	Unknown	4 (4.7)	6 (3.1)

Mean age	Mean (sd)	39.6 (9.7)	44.9 (5.9) *

Mean age at uptake of smoking	Mean (sd)	20.8 (6.5)	22.4 (8.6)

Number of years in Netherlands	Less than26 years	n.a.	98 (50.3)

	More than 26 years	n.a.	97 (49.7)

Contacts in leisure time	Most Dutch	n.a.	12 (6.1)

	Both	n.a.	60 (30.6)

	Most Surinamese	n.a.	67 (34.2)

	Only Surinamese	n.a.	56 (28.6)

*p < 0.01 **p < 0.001

### Smoking related beliefs

The women who smoked in the Surinamese sample and the Dutch sample differed regarding most of the attitudinal beliefs (Table 3). Surinamese women in the Netherlands felt more comfortable, were less ashamed of themselves and believed smoking to be sociable. Furthermore, the women in Suriname thought more often that their smoking was a nuisance to other people or that they had a higher chance of getting heart disease. With respect to the beliefs about subjective norms, the subjective norm of partner and of friends (male and female) differed significantly between both samples. Surinamese women in the Netherlands perceived less disapproval about their smoking behaviour from their partner and their friends than their counterparts in Suriname. Regarding perceived behaviour, only the number of female friends who smoked differed; more Surinamese women in the Netherlands had female smoking friends. No differences have been found regarding the subjective norm among family members and perceived smoking behaviour of family members.

**Table 3:** Differences in beliefs towards smoking among Surinamese women in the Netherlands and Suriname

	Suriname		Netherlands	
	**n**	**% agreed**	**n**	**% agreed**

**Affective attitude**				

If you smoke do you feel comfortable?	37	(43.5)	125	(63.8)*

If you smoke do you feel normal?	55	(64.7)	136	(69.4)

If you smoke are you ashamed of yourself?	25	(29.4)	28	(14.3)*

Smoking is sociable	43	(49.4)	130	(66.3*)

**Cognitive attitude**				

If I smoke I have a higher chance of getting heart disease	75	(88.2)	152	(77.9)

If I smoke I bother other people	77	(90.6)	150	(76.9)*

If I smoke I have a higher chance of getting lung disease	80	(94.1)	150	(76.9)*

		% approved		% approved

**Subjective norm partner**				

What does your partner think about your smoking behavior?	31	(36.5)	150	(76.5)*

**Subjective norm of family**				

What do the men in your family think about your smoking behavior?	39	(45.9)	122	(62.2)

What do the other women in your family think about your smoking behavior?	34	(40.0)	98	(50.0)

**Subjective norm of friends**		% approved		% approved

What do your male friends think about your smoking behavior?	50	(58.8)	82	(82.1)*

What do your female friends think about your smoking behavior?	45	(52.9)	75	(75.0)*

		% agreed		% agreed

**Perceived behavior partner**				
Does your partner smoke?	42	(49.4)	57	(29.2)

**Perceived behavior of family**				

Do your male family members smoke?	70	(82.4)	145	(74.4)

Do your female family members smoke?	45	(52.9)	58.7	(58.7)

**Perceived behavior of friends**				

Do your male friends smoke?	58	(68.2)	138	(70.4)

Do your female friends smoke?	41	(48.4)	141	(72.3)*

*p < 0,01

Table 4 presents the odds ratios of the attitudinal and social influence scales from women who smoked in the Dutch sample compared to those who smoked in the Surinamese sample, adjusted for age, educational level, ethnicity and religion. Compared to women in Suriname, Surinamese women in the Dutch sample had approximately three times more often a positive affective attitude towards smoking. Moreover, three times more often they didn't perceive any health consequences of smoking. Surinamese women in the Netherlands perceived six times more often a positive norm of partner toward smoking than the Surinamese women in Surinam. Furthermore, three times as many women in the Dutch sample perceived a positive norm of friends compared to the Surinamese sample, which was statistically significant.

**Table 4:** Odds Ratios of determinants in the Dutch sample (SUNSET) compared to those in Surinamese sample (CVRFO), adjusted for age, ethnicity, education and religion

	OR	95% CI
Positive affective attitude towards smoking	2.59	(1.05; 6.39)

Perceived no negative consequences of smoking	3.33	(1.31; 8.41)

Positive subjective norm of partner	6.49	(2.71; 15.56)

Positive subjective norm of family towards smoking	1.91	(0.89; 4.14)

Positive subjective norm of friends towards smoking	3.30	(1.50-7.25)

Perceived behavior of partner: smoker	1.70	(0.79-3.63)

Perceived behavior of family: half or more smokers	0.56	(0.27-1.16)

Perceived behavior of friends: half or more smokers	1.88	(0.88; 4.01)

OR: odds ratio CI: confidence interval

## Discussion

This study aims to provide more insight into the psychosocial determinants of smoking among Surinamese migrant women in the Netherlands and Surinamese women in Surinam. Of the female Surinamese smokers, those in the Netherlands were three times more likely to perceive emotional and cognitive advantages of smoking than those in Suriname. These women also perceived a positive norm of their partner and among friends regarding smoking more frequently.

As far as we know this is the first study in which the differences between the underlying psychosocial determinants between migrants and their counterparts in the country of origin have been explored. Other studies that have focused on psychosocial determinants in the context of migration, look at the changes by assessing the associations of *acculturation *with health related behaviour and its determinants of immigrants in the host country, but did not compare these data with any data on people in the country of origin [23-25].

Some limitations of this study need to be considered before discussing the results. Firstly, due to the cross-sectional design we are not sure whether migrant women changed their smoking behaviour after migration. It is possible that the women who migrated differ regarding smoking behaviour and beliefs about smoking from those who stayed in Suriname. However, a previous study suggests that Surinamese women in the Netherlands are in an earlier stage of the tobacco epidemic than the ethnic Dutch women [3]. This might be an indication that the smoking pattern in Surinam will be in a previous stage as well, implying lower smoking rates. Nevertheless, longitudinal studies among recently migrated people should test whether the differences we have found are really the results of migration.

Secondly, we were not able to include non-smoking women in Suriname in our study, which implies that we cannot be sure that the determinants account for the differences in smoking prevalence in Surinamese women in both countries. However, as many studies have shown that these determinants are important predictors of behavioural change, our study seems to hold strong indications that the differences in determinants might contribute to the differences in smoking behaviour.

Despite these limitations, our study found interesting results regarding the psychosocial determinants of smoking among female Surinamese migrants. As expected we found a higher prevalence of smoking in female Surinamese migrants in the Netherlands compared to those in the country of origin. The relatively positive attitudes and norms are in line with the differences in the prevalence. Explanations for these results can be found in the different place where the women live.

In Western countries, smoking is culturally less restricted for women than in non-Western countries such as Suriname. In contrast to smoking in Suriname, in the Netherlands smoking is not regarded as shameful for women and they can smoke where they want. This seems to be reflected in the higher percentage of smokers among Surinamese women in the Netherlands. However, there is also more anti-smoking prevention available in Western countries, which might prevent a positive attitude among these women [26]. Though, Surinamese women in the Netherlands might be less convinced about the negative consequences of smoking than their counterparts in Surinam. This may indicate that these prevention activities do not adequately reach the Surinamese population.

However, when we compare results of a study among Dutch students with those of the Surinamese women, we have found that the percentage of Dutch students that were aware of negative consequences of smoking for heart disease was similar with or even lower than the percentage we have found (71.2% vs. 77.9% in our study) [27]. This may indicate that, despite all prevention activities, the attitude of the general population is relatively positive, and that the attitude of the Surinamese women in the Netherlands seems to converge to this attitude.

Regarding the subjective norm of family and the number of family members who smoke we have found no differences between the women in both countries. This might indicate that the norms among the family did not change after migration. It may be that, due to migration, family ties are loosening and that as a result norms in the family have less influence on smoking behaviour. The increase in smoking may be more associated with the increase of friends who smoke and a positive subjective norm towards smoking among friends and the partner in combination with a positive attitude towards smoking. As a result Surinamese women in the Netherlands are more exposed to a proximal pro-smoking social environment compared to the women in Surinam.

The differences observed should be understood in the broader context of the changes that people experience after migration and which are also known to contribute to health inequalities. These include cultural changes, changes in socio-economic position, social position and perceived discrimination [8]. With regard to smoking behaviour, previous research shows for example that socio-economic position is an important factor in relation to migration and smoking behaviour. As the Surinamese migrant women have a relatively low socio-economic position they more frequently live in disadvantaged neighbourhoods where smoking norms are more positive [3,8,9,28]. Also, their relative disadvantaged position may be related to a lower access of health care and (smoking) prevention programs [29].

With regard to other contextual factors, the results of our study will be best generalisable to other migrant women who have a similar background. Regarding the Surinamese population, their colonial background, a fluency of the Dutch language and a lower socio-economic position than the ethnic Dutch are important contextual characteristics. Hence, our results may be most generalisable to migrant female groups with a colonial background from other countries, such as South Asians in the UK. However, as we see also increases in smoking behaviour among other migrant populations, similar changes might be present in other migrant groups as well.

The results of this study indicate that anti-smoking interventions for migrant women should focus on the negative consequences of smoking and the prevention of a positive norm towards smoking - for example, by developing culturally targeted interventions using role modelling [30]. These interventions should be developed for other female migrants from non-Western countries as well as we see a similar increase in smoking prevalence either following migration, or as integration increases.

## Conclusions

In conclusion, the results of this study suggest that migrant women are more positive towards smoking and perceived a more positive norm towards smoking when compared with women in the country of origin. Interventions targeted at the psychosocial determinants regarding smoking for newly migrated women, in particular the consequences of smoking and the norm towards smoking might help to prevent an increase in smoking in those populations.

## List of Abbreviations

CVFRO: CardioVasculair RisicoFactor Onderzoek; SUNSET: SUrinamers in Nederland, Studie naar gezondheid en Etniciteit; OR: odds ratio

## Competing interests

The authors declare that they have no competing interests.

## Authors' contributions

VN contributed to conception and the design, analysis and interpretation of the data and has written main parts of the manuscript, MVvdP contributed importantly to the conception and the design, carried out the additional data collection and the analysis contributed to the interpretation of the data and has been involved in drafting the manuscript, MYvE contributed to the design of the CVFRO study and the data collection and has been involved in drafting the manuscript. KS contributed to the conception and design of the SUNSET study and the additional data collection, the interpretation of the data and has been involved in drafting the manuscript. All authors approved en read the final manuscript.

## Box 1

In 1975, almost half the population of the former Dutch colony Surinam migrated to the Netherlands. Approximately 80% of these Surinamese immigrants in the Netherlands are Hindustani ('South Asian', originally from the Indian sub-continent) or African (mixed African, Indian and European, but predominantly of African origin). Other ethnicities include Chinese and Javanese.

## Pre-publication history

The pre-publication history for this paper can be accessed here:

http://www.biomedcentral.com/1471-2458/11/397/prepub
